# Effect of Drugs Used in Pharmacotherapy of Type 2 Diabetes on Bone Density and Risk of Bone Fractures

**DOI:** 10.3390/medicina60030393

**Published:** 2024-02-26

**Authors:** Agnieszka Wikarek, Małgorzata Grabarczyk, Katarzyna Klimek, Agata Janoska-Gawrońska, Magdalena Suchodolska, Michał Holecki

**Affiliations:** 1 Student Scientific Society at the Department of Internal Medicine, Autoimmune and Metabolic Diseases, Faculty of Medical Sciences in Katowice, Medical University of Silesia, 40-055 Katowice, Poland; 2 Department of Internal Medicine, Autoimmune and Metabolic Diseases, School of Medicine, Medical University of Silesia, 40-055 Katowice, Poland

**Keywords:** osteoporosis, diabetes mellitus, antihyperglycemic drugs, bone metabolism

## Abstract

This review summarizes the complex relationship between medications used to treat type 2 diabetes and bone health. T2DM patients face an increased fracture risk despite higher bone mineral density; thus, we analyzed the impact of key drug classes, including Metformin, Sulphonylureas, SGLT-2 inhibitors, DPP-4 inhibitors, GLP-1 agonists, and Thiazolidinediones. Metformin, despite promising preclinical results, lacks a clear consensus on its role in reducing fracture risk. Sulphonylureas present conflicting data, with potential neutral effects on bone. SGLT-2 inhibitors seem to have a transient impact on serum calcium and phosphorus, but evidence on their fracture association is inconclusive. DPP-4 inhibitors emerge as promising contributors to bone health, and GLP-1 agonists exhibit positive effects on bone metabolism, reducing fracture risk. Thiazolidinediones, however, demonstrate adverse impacts on bone, inducing loss through mesenchymal stem cell effects. Insulin presents a complex relationship with bone health. While it has an anabolic effect on bone mineral density, its role in fracture risk remains inconsistent. In conclusion, a comprehensive understanding of diabetes medications’ impact on bone health is crucial. Further research is needed to formulate clear guidelines for managing bone health in diabetic patients, considering individual profiles, glycemic control, and potential medication-related effects on bone.

## 1. Introduction

The prevalence of diabetes is increasing, with as many as 422 million adults suffering from the disease, according to the World Health Organization (compared to 108 million in 1980) [1]. The risk of developing type 2 diabetes (T2DM) as well as osteoporosis both increase with age [2]. It has become widely accepted that diabetes (both T1DM and T2DM) has a direct impact on bone metabolism, with fragility fractures representing an often-underestimated consequence [3,4]. It is important to bear in mind that extending the life expectancy of these patients will increase the global burden of both diseases with fragility fractures that have a huge impact on morbidity and mortality. Interestingly, in T2DM individuals, bone fractures occur at higher bone mineral density (BMD) values, and the T-score is often above the osteoporotic range. A disturbed bone microarchitecture is observed, which results in a decrease in bone strength to loads and stresses [5]. There is an increased risk of hip fractures, with BMD higher by 0.4 and 0.6 SD in men and women, respectively. The Trabecular Bone Score (TBS) is considered a better predictor of bone fractures than BMD [6].

**Figure 1:**
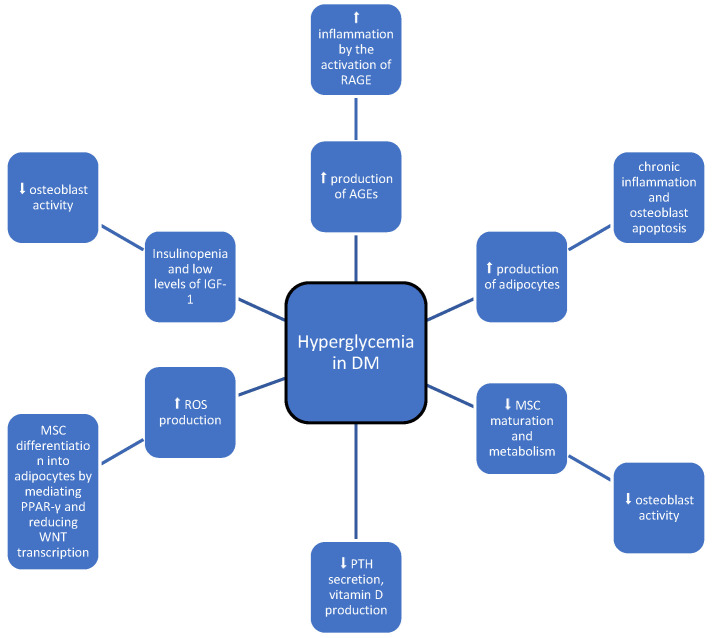
Possible impact hyperglycemia in type 2 diabetes on bone metabolism; symbols: ⬆—increase, ⬇—decrease.

Patients with diabetes have a 32% increased risk of any fracture compared to the general population (relative risk [RR] 1–32, 95% CI 1-17-1-48). T2DM increases this risk by (1–22, 1-13-1-31), respectively. The risk of fractures varies by location; for hip fracture, the risk was (1–27, 1-16-1-39). When analyzing the risk of fractures by gender, men had a correspondingly higher fracture risk (RR 1–90, 95% CI 1-30-2-58) compared to women (1–44, 1-19-1-70). Moreover, patients with obesity are at particular risk of fractures [7]. The pathophysiological changes in T2DM affecting bone metabolism are complex and dependent on many factors, including muscle-derived hormones, inflammatory cytokines, hydrogen sulfide, and incretin levels. In addition, the secretion of cortisol, its activation, and the sensitivity of target cells play an important role (Figure 1). All of the above-mentioned factors affect bone formation and resorption and both collagen production and bone marrow adiposity. Together, they reduce bone strength by altering its microarchitecture. Another important factor that increases the risk for fractures is propensity for falling, which is higher in individuals suffering from frailty syndrome, micro and macroangiopathic complications, and drug-induced hypoglycemia. Risk factors also include advanced age, vision impairment, impaired balance, peripheral neuropathy, comorbidities, a higher body mass index, and musculoskeletal disorders. Drug-induced hypoglycemia should be avoided, as it is not only associated with a higher risk of falls, but also cardiovascular complications and cognitive impairment. 

Maintaining tight glycemic control should be discouraged in elderly patients with multiple cardiovascular complications. All of these risk factors should be evaluated and addressed properly. Environmental hazard modification, proper visual assessment, withdrawal of psychotropic medication, and hypoglycemia and hypotension avoidance are of great importance.

Furthermore, antidiabetic medications affect bone metabolism in specific ways (Table 1 and Table 2) [6].

Patients with T2DM should follow general non-pharmacological recommendations for the prevention of osteoporosis such as lifestyle modification, including regular and adequate physical activity, smoking cessation, alcohol restriction, and a diet with proper calcium and vitamin D intake. These guidelines do not differ from recommendations for non-diabetics [8]. In addition, rapid weight loss is not recommended, as it has a negative effect on bone fractures [9]. Global recommendations for the management of hyperglycemia in type 2 diabetes suggest a holistic approach and individualization of therapy depending on the patient’s profile. This includes managing blood glucose, weight, cardiovascular risk factors, comorbidities, and complications. The main goals of reducing risk fracture are to maintain optimal glycemic control to avoid hypoglycemia and to manage comorbidities appropriately. There is a lot of emphasis on possible renal, cardiovascular, and other benefits of various antidiabetic agents. There is also a link between diabetes, antidiabetic treatment, and their influence on bone loss and structure. The aim of this article was to analyze the possible effects of antidiabetic drugs on bone metabolism and the risk of bone fractures.

## 2. Methodology

A systematic literature search for studies was conducted in electronic databases (PubMed, Embase, Cochrane) using combinations of the key terms “type 2 diabetes” or “diabetes mellitus” or “anti-diabetic drugs (each one separately)” and “osteoporosis” or “bone metabolism” or “fracture” or “diabetoporosis”. Each author conducted an independent search and the results were compiled. Special attention was paid to work on the management of patients with T2D and osteoporosis. The articles included in the review were published in English and available until June 2023. 

## 3. Discussion

### 3.1. Metformin

**Figure 2:**
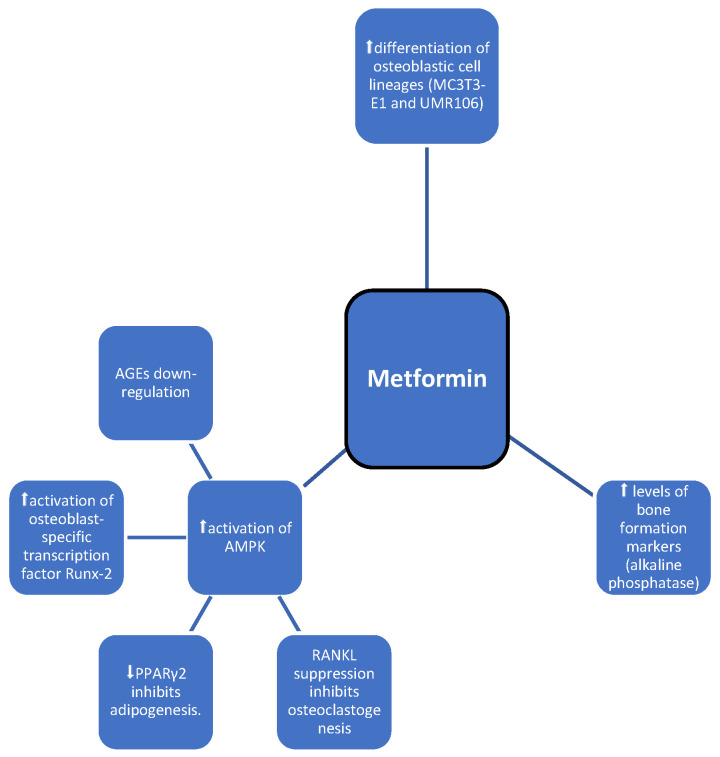
Possible impact of metformin on bone metabolism; symbols: ⬆—increase, ⬇—decrease.

Metformin is a first line oral antidiabetic drug that improves cellular insulin sensitivity in insulin-resistant individuals, especially those with type 2 diabetes. There is substantial evidence to suggest that metformin has beneficial effects on the maintenance of bone metabolism [10,11]. It has been shown that metformin has a positive effect on BMD in preclinical studies [5]. Metformin has shown its regulatory effects on AMPK to reduce osteoclastogenesis. Metformin affects glucose metabolism (Table 1, Figure 2) through the activation of AMP-activated protein kinase (AMPK). AMPK is expressed in bone cells and has subunits differentiated for expression and activation. AMPK α1 is expressed in primary osteoblasts, primary bone marrow macrophages, osteoclasts, and other bone cell lines [12]. Metformin affects the differentiation and mineralization of osteoblastic MC3T3-E1 cells through AMPK and nitric oxide synthesis and the production of bone morphogenetic protein-2 [13]. In another study, Cortizo et al. showed an effect of metformin on the differentiation of osteoblastic cell lineages (MC3T3-E1 and UMR106), in addition to increased levels of bone formation markers such as alkaline phosphatase [14]. What is more, metformin has the ability to prevent AGE-induced changes, i.e., the induction of apoptosis, caspase-3 activity, reduction of RAGE activity, and changes also involved in the reduction of intracellular oxidative stress. Although the direct mechanisms of metformin signaling are not fully understood, data indicate AGE-RAGE interaction in modulating osteoblastic cell growth and differentiation [15]. In addition, metformin has an osteogenic effect, which is due to an increase in the osteoblast-specific transcription factor Runx2/Cbfa [16]. Thus, evidence suggests that metformin has a direct effect on inhibiting bone loss. A population-based cohort study conducted in South Korea found no association between bone fracture risk and metformin use in patients with T2DM. Oh T. et al. also showed no clinical benefit in terms of bone fracture risk in patients with T2DM [17]. In contrast, Vestgard et al. showed a reduced risk of bone fractures [18]. In addition, subsequent studies, including two meta-analyses, have shown that metformin use was associated with a reduced risk of total bone fractures among patients with diabetes. The discrepancy between those observations may be due to differences in considered populations, experimental methods, concentrations, and duration of treatment with metformin. Thus, current evidence that metformin therapy reduces fracture risk is lacking [19]. Therefore, further studies are needed to investigate possible beneficial effects of metformin on bone metabolism to obtain a clinical consensus. Overall, metformin seems to be an optimal choice among diabetic individuals at high risk of fragility fractures in the absence of standard contraindications. 

### 3.2. Sulphonyloureas

**Figure 3:**
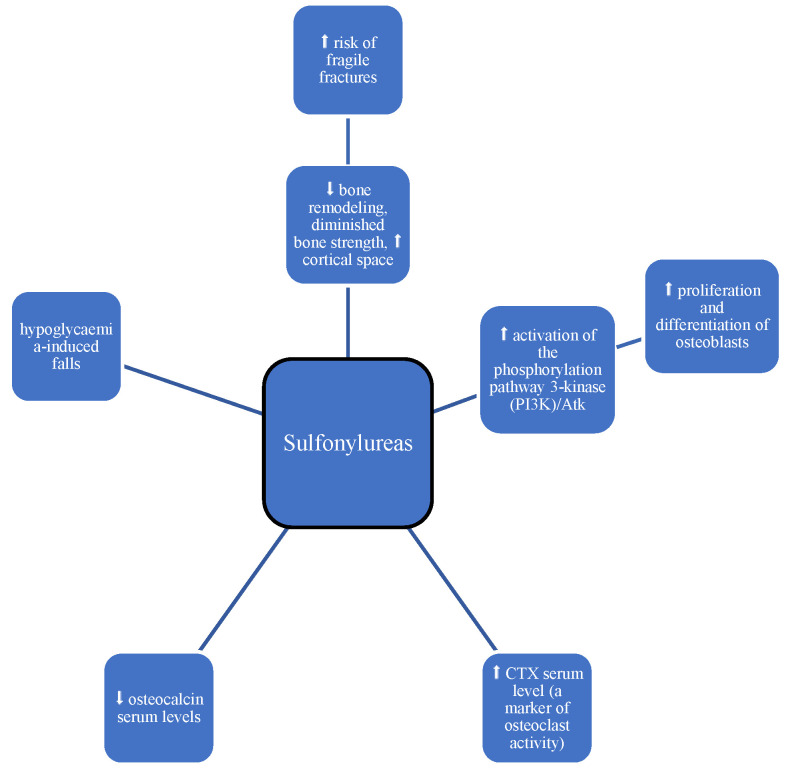
Impact of SUs on bone metabolism; symbols: ⬆—increase, ⬇—decrease.

Sulphonylureas (SUs) are widely used in patients with T2DM, but the data on their effects on bone metabolism are limited. Ma et al. demonstrated the effect of glimepiride (a third-generation sulphonylurea) on enhancing the proliferation and differentiation of rat osteoblasts through activation of the phosphorylation pathway 3-kinase (PI3K)/Atk (Table 1, Figure 3). In addition, there is the possibility of reducing the adverse effect of hyperglycemia on the osteoblast [20]. However, further human studies provided no data supporting a beneficial effect of SUs on bone remodeling or on measures of bone mineral density (BMD) [21,22]. 

On the other hand, SUs can lead to hypoglycemia-induced falls with subsequent bone fractures. However, the results of studies are not consistent on whether there is a direct correlation between SU use and general risk of falls and fall-associated fractures [23,24].

It has been suggested that they have a neutral effect on bone [25]. In their study, Vestergaard et al. showed a reduced risk of bone fractures (adjusted OR, 0.88; 95% CI, 0.80–0.96) during sulfonylurea use. Reduced risk of hip fractures was also observed (adjusted OR, 0.77; 95% CI, 0.63–0.95) [18]. On the other hand, Monami et al. showed no statistically significant association between sulfonylurea treatment and fracture risk. However, this risk was reduced in patients using sulfonylurea (adjusted OR, 0.77; 95% CI, 0.44–1.37) [13]. Similar results were observed by Zhang YS et al. [26]. The ADOPT study was conducted in both men and women using sulfonylurea and showed a reduction in CTX serum level (a marker of osteoclast activity) [21]. On the other hand, a study by Rico H. et al. showed reduced osteocalcin serum levels among sulfonylurea-treated patients [27]. Acknowledgements from the most recent meta-analysis suggest that sulfonylurea use is associated with a 14% increase in fracture risk in patients with diabetes. The risk was considered similar to that of thazolidendione but lower than that of insulin [20,28]. To sum up, there are currently few preclinical and clinical studies available on the effects of sulfonylurea on bone metabolism, but the majority of them concluded that sulfonylureas have at least a neutral effect on bone metabolism. However, further confirmation is required to determine whether the observed association between sulphonylurea use and fracture risk is due to SU treatment itself or confounding factors. Sulfonylureas should be used with caution, especially in the elderly [29], and should be avoided in individuals prone to hypoglycemia. 

### 3.3. SGLT-2 Inhibitors

**Figure 4:**
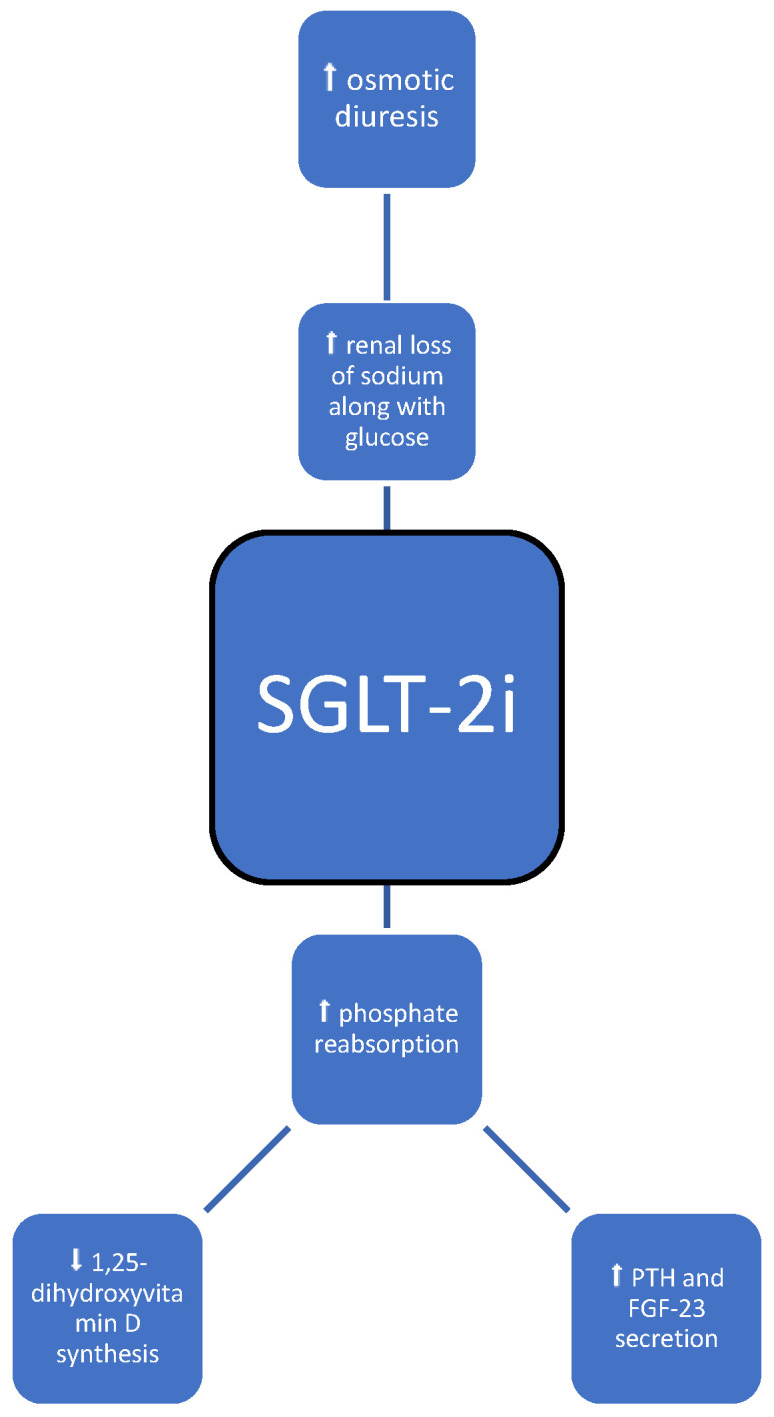
Possible impact of SGLT-2i on bone metabolism; symbols: ⬆—increase, ⬇—decrease.

SGLT-2 inhibitors, a relatively new group of drugs primarily used in patients with type 2 diabetes (Table 1, Figure 4), may have a transient impact on calcium and phosphorus homeostasis. SGLT-2 inhibition promotes phosphate reabsorption in the proximal tubule (sodium–phosphate cotransport) to compensate for renal loss of sodium along with glucose. An initial increase in serum phosphate has been described [30], and it resolves after 3 months of therapy. After a temporal increase in phosphate absorption, there is an increase in PTH and FGF-23 secretion and then inhibition of 1,25-dihydroxyvitamin D synthesis; consequently, there is reduced intestinal phosphate absorption and increased renal phosphate excretion by the kidney. This might be the reason for the normalization of phosphate serum levels after 3 months [31,32]. Lower levels of 1,25-dihydroxyvitamin D suggest domination of the FGF-23-mediated mechanism over the PTH-mediated phosphaturia. Similarly, this observation was confirmed in a study by de Jong M.A. et al., who found that PTH and FGF-23 levels increased by 15% and 20%, respectively [33]. 

Last but not least, theoretically SGLT-2 inhibitors may predispose to dehydration [34], as they cause osmotic diuresis and intravascular volume contraction, orthostatic hypotension [34], and increased risk for falls, thus increasing the overall risk of fractures. However, the FAERS [35] (the real-world safety profile of sodium–glucose co-transporter-2 inhibitors among older adults (≥75 years): a retrospective, pharmacovigilance study) study showed that SGLT-2-i therapy was not associated with increased cases of hypotension, falls, and syncope. In the FAERS study, a borderline significance in the increased numbers of fractures, with no significant differences between age groups or specific flozins, was found, and the researchers concluded that they did not identify a robust safety signal of fractures [35]. 

Therefore, caution must be taken when prescribing these drugs to the elderly, patients with renal impairment or low systolic blood pressure, and those on diuretics [36]. 

All of the metabolic disturbances can possibly affect bone metabolism and risk of fractures, but the available literature does not indicate an evident relationship between the use of flozins and fractures. In the meta-analysis of 27 randomized controlled trials that compared the efficacy and safety of SGLT-2-i to a placebo in 20,895 diabetes mellitus type 2 patients, with an average study duration time of 64.22 weeks, the relative risk of fracture was 1.02 (95% CI [0.81, 1.28]), with low heterogeneity. Different SGLT-2-i dosages were used, and treatment was not correlated with a higher risk of fracture. Also, three trials with 1303 patients reported a change in the bone mineral density (BMD) from baseline, but when compared with the results of the placebo groups, the BMDs in the SGLT-2 inhibitor groups did not decrease the BMD measured at the lumbar spine, femoral neck, total hip, and distal forearm [37]. 

In a study conducted by List et al., treatment with dapagliflozin resulted in no significant alteration from baseline in serum calcium, 1,25-dihydroxyvitamin D, and 25-hydroxyvitamin D levels. Also, changes in the 24-h urinary calcium-to-creatinine ratio were similar to the placebo [38]. Dapagliflozin treatment was found to have no impact on the bone mineral density and bone formation and resorption markers after 50 weeks of treatment in both male and post-menopausal female patients [39]. 

On the other hand, in a meta-analysis of 78 randomized controlled trials, for all flozins, treatment with canagliflozin alone was associated with a higher incidence of fracture [40]. The CANVAS (CANagliflozin cardioVascular Assessment Study Program) study revealed a higher risk of low-trauma fracture and all fracture in the canagliflozin group than in the placebo group, but the CANVAS-R study did not confirm this observation. So far, there is no obvious explanation for the differences between the two trials, which included comparable patient groups and assessed the same intervention [41,42]. The reason for the increased risk of fractures with canagliflozin remains unknown [43]. 

A long-term follow-up study of fracture rates during treatment with flozins, especially with canagliflozin, is needed, as the results of studies are unclear and require further investigation [40,43]. Regarding bone metabolism, it has been shown that canagliflozin might exert negative effects on bone density, bone resorption, and fracture risk at the hip. Dapagliflozin and empagliflozin on the other hand have not been shown to have a significant impact on BMD, bone markers, or fracture risk, with rather neutral effects on bone health. However, the concerns raised from studies with canagliflozin affected the whole class. Further studies are needed to elucidate the mechanisms of bone loss and the real safety profile among these newly used medications. To sum up, treatment with SGLT2 inhibitors is not significantly associated with an increased risk of fractures, and canagliflozin should be used with caution, as concerns have been raised about potential harmful effects on bone health. 

### 3.4. DPP-4 Inhibitors

**Figure 5:**
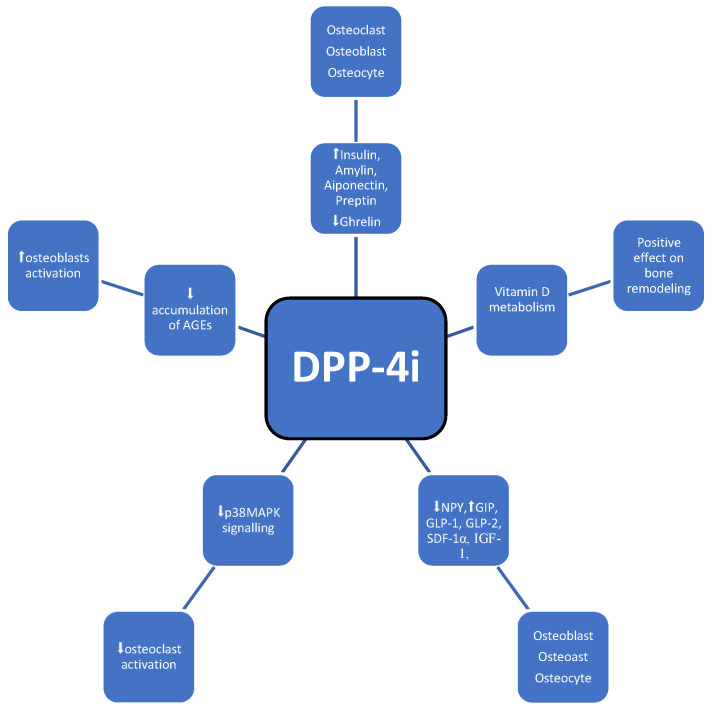
Possible impact of DPP-4i on bone metabolism; symbols: ⬆—increase, ⬇—decrease. Abbreviations: GIP—gastric inhibitory polypeptide, GLP-1—including glucagon-like peptide 1, GLP-2—including glucagon-like peptide 2, IGF-1—insulin-like growth factor 1, SDF-1α—stromal cell-derived factor-1, NPY—neuropeptide Y, AGE—advanced glycation end products.

Dipeptidyl peptidase-4 (DPP-4) is a widely expressed serine protease that selectively cleaves alanine and proline from polypeptide substrates, inactivating these substrates, including glucagon-like peptide 1 (GLP-1) and gastric inhibitory polypeptide (GIP). DPP-4 inhibitors function by blocking the inactivation of GIP and GLP-1 modulate glucose-induced insulin secretion (Table 1, Figure 5) [44].

The impact of dipeptidyl peptidase 4 inhibitors (DPP-4-i) on bone metabolism is complex and multidirectional and has been widely described by Yinqiu Yang et al. First of all, they affect bone metabolism through their substrates (increase in GIP, GLP-1, GLP-2, IGF-1, SDF-1α, and a decrease in NPY) and through a vitamin D-linked pathway, which induces bone growth and bone remodeling (through the absorption and activation of vitamin D; the decrease in adipose tissue inflammation through a reduction in the levels of inflammatory cytokine expression and consequent inflammation-induced bone resorption; and the decrease in AGE-receptor gene expression) [44,45]. AGE accumulation or AGE/RAGE (advanced glycation end products/advanced glycation end product receptors) imbalance directly decreases the number and function of osteoblasts [28]. Also, the impact is mediated through DPP-4-related energy metabolism via the increase in insulin, adiponectin, amylin, and preptin and the decrease in ghrelin and p38 mitogen-activated protein kinase, which results in lower osteoclasts formation [44,45]. The most recent results from a meta-analysis conducted by Lili Huang et al. indicate an increase in BMD and a reduction in the risk of osteoporosis among patients treated with DPP-4-i [46]. 

This class of drugs is associated with a lower risk of fractures. For example, sitagliptin, a strong and highly selective DPP-4 inhibitor, improves bone mineral density and bone quality and was positively correlated with bone formation markers such as alkaline phosphatase and osteocalcin [44]. These observations have been confirmed in numerous clinical studies, including RCTs. Monami et al., in a meta-analysis of 28 RCTs, reported that DPP-4-i treatment reduced fracture risk when compared to placebo or other anti-diabetic medications (Mantel–Haenszel-odds ratio [MO-OR] 0.60, 95% confidence interval [CI] 0.37–0.99, *p* = 0.045), and the MH-OR for DPP-4 inhibitors treatment was 0.54 (0.28–1.03, *p* = 0.063) and 0.70 (0.32–1.52, *p* = 0.37) in trials with a duration <52 weeks or ≥52 weeks, respectively, and seven ≥52 weeks trials were available. Therefore, the positive effect of DPP-4-I on bone health appears to be dependent on treatment duration, as it was more strongly expressed when the duration time was ≥52 weeks [47].

Similar results were found in a different study by Dombrowski et al., where patients treated with DPP-4-i along with metformin had a lower fracture risk than those treated only with metformin [48]. In a retrospective nationwide South Korean cohort, subjects treated with a combined therapy of metformin and DPP4-I showed a lower non-vertebral fracture risk (HR = 0.82, *p* = 0.086) after adjusting for all confounding variables [49]. In a 2021 South Korean nationwide population-based retrospective cohort study, the risk of bone fracture was not different between groups treated with DPP-4-i and SGLT-2-i [50]. In most studies, DPP-4 inhibitor use was inversely associated with fracture risk. The beneficial effect of DPP-4 inhibitors on bone health in diabetic individuals provides an additional advantage of these antihyperglycemic agents beyond their glucose-lowering and metabolic effects. 

### 3.5. GLP-1 Agonists

**Figure 6:**
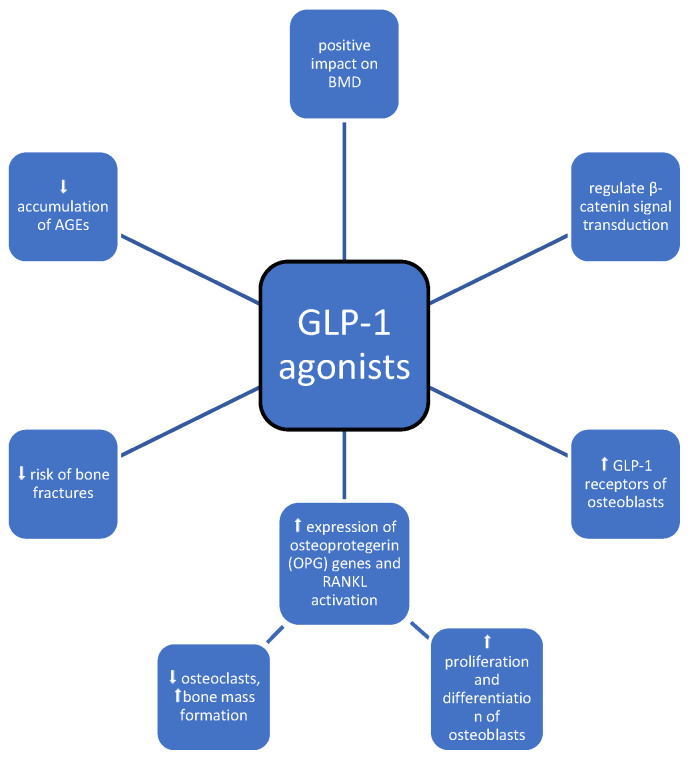
Impact of GLP-1 agonists on bone metabolism; symbols: ⬆—increase, ⬇—decrease.

GLP-1 is an intestinal peptide produced by intestinal epithelial L cells, the secretion of which is induced by an increase in serum glucose concentration and the consumption of meals (Table 1, Figure 6). GLP-1 agonists increase insulin synthesis and secretion and inhibit appetite [51]. These novel antidiabetic drugs are considered to have a positive impact on bone health [51,52] as they reduce the accumulation of advanced glycation end products (AGEs) [53], stimulate GLP-1 receptors of osteoblasts [54,55], regulate β-catenin signal transduction [56], and increase the expression of osteoprotegerin (OPG) genes, which affects the OPG/nuclear factor-κB ligand-receptor activator (RANKL)/nuclear factor-κB receptor activator (RANK) pathway, inducing the activation, proliferation, and differentiation of osteoblasts, the inhibition of osteoclasts, and bone mass formation [57]. These assumptions were clinically confirmed by an assessment of BMD in patients receiving GLP-1 agonists compared to patients receiving placebo [52]. Exenatide and dulaglutide had a positive impact on BMD [51]. In another study, when compared with placebo and other anti-diabetic drugs, GLP-1 agonist treatment (liraglutide and lixisenatide) was associated with a significant reduction in the risk of bone fractures (ORs, 0.56; 95% CI, 0.38–0.81 and 0.55; 95% CI, 0.31–0.97, respectively), and the positive effects were dependent on the duration of treatment [51]. 

Emerging data suggest that GLP-1RAs and their analogs have a positive impact on skeletal metabolism by promoting bone formation and inhibiting its absorption. Some studies have shown that GLP-1RAs have anabolic effects on bone metabolism, but the specific molecular mechanisms are still unclear. Based on clinical data, these novel drugs have been shown to treat hyperglycemia without the risk of hypoglycemia and promote weight loss without an elevated risk of fractures. Therefore, this class of drugs is considered an effective option for diabetic patients with osteoporosis and those at higher risk for bone disorders. 

### 3.6. Thiazolidinediones

**Figure 7:**
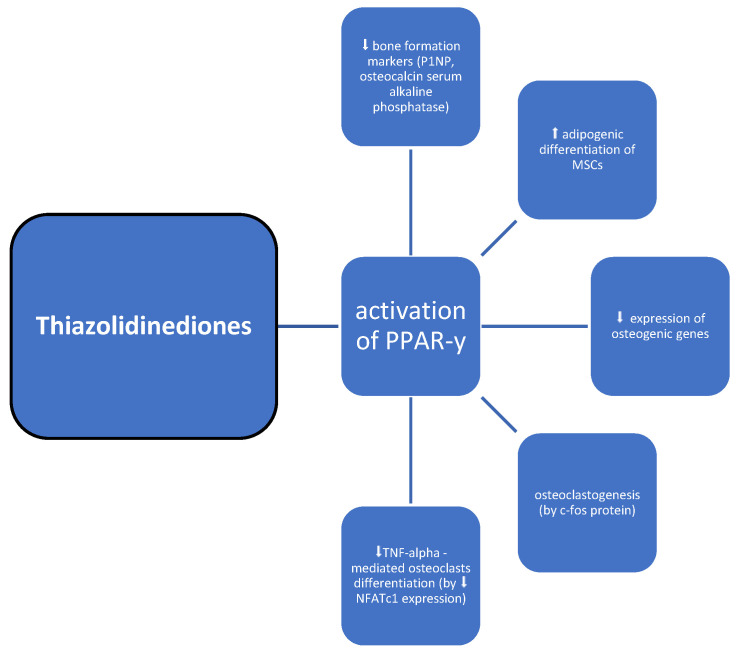
Possible impact of thiazolidinediones on bone metabolism; symbols: ⬆—increase, ⬇—decrease.

Thiazolidinediones (TZDs) are oral hypoglycemic drugs which act as insulin sensitizers [58]. The molecular mechanism underlying the pharmacological effects is based on the activation of the intranuclear peroxisome proliferator-activated receptor (PPAR)-γ, regulating the expression of multiple genes involved in adipocyte differentiation, inflammation, lipid metabolism, and glucose control (Table 1, Figure 7) [59]. A PPAR-γ presence was found in pluripotent mesenchymal stem cells (MSCs), which among others can differentiate into osteoblasts and marrow adipocytes [60]. The activation of PPAR-γ determines the direction of MSC differentiation by shifting the balance between bone formation and adipogenesis. Therefore, through these receptors, TZDs induce adipogenic differentiation of stromal cells at the expense of osteoblastogenesis and decrease the expression of osteogenic genes and markers. This imbalance can ultimately result in bone loss [61]. This effect can be enhanced by PPAR-y stimulated osteoclastogenesis via direct regulation of c-fos protein [62]. On the other hand, some studies suggest otherwise—TZDs down-regulate NFATc1 expression, therefore inhibiting TNF-alpha-mediated osteoclast differentiation and further resorption [62,63].

In humans, an analysis of 200 patients with T2DM revealed a significant decrease in BMD at the spine and hip among patients using glitazones [61]. The results of a meta-analysis of 22 randomized controlled trials indicate that pioglitazone treatment is associated with a significant increase in the incidence of fractures in females [64]. A study conducted on a group of healthy postmenopausal women showed that a 14-week treatment with rosiglitazone resulted in a significant reduction in BMD, a reduction of bone formation markers—P1NP, osteocalcin, and serum alkaline phosphatase—the inhibition of bone formation, and the acceleration of bone loss [65]. Schwartz et al. presented that the duration of TZD therapy among diabetic patients was associated with greater bone loss [66]. On the other hand, these outcomes were not supported among the population of men [66]. Moreover, bone mass loss caused by TZDs may be irreversible [67]. Despite the positive metabolic results of TZD therapy in diabetic patients, the possible adverse effects on bone metabolism should be considered, especially in patients with an initial high risk of fractures. Therefore, they are contraindicated in osteoporosis, and we believe that they should be avoided in populations at risk of bone disorders.

### 3.7. Insulin

**Figure 8:**
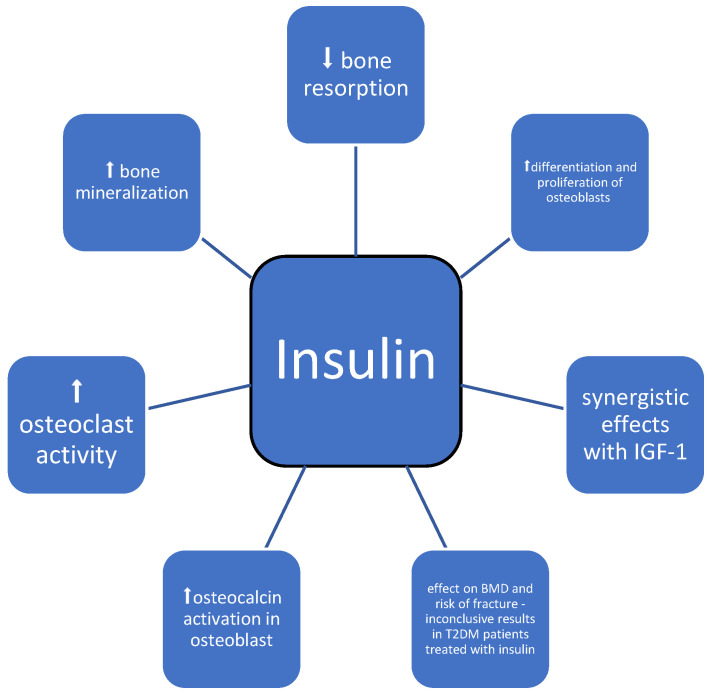
Possible impact of insulin on bone metabolism; symbols: ⬆—increase, ⬇—decrease.

Insulin presents an anabolic effect on BMD—it promotes the differentiation and proliferation of osteoblasts [68,69]. The injection of insulin in adult mice resulted in increased bone mineralization and the inhibition of bone resorption [70]. Furthermore, in type 1 diabetes, the lack of insulin was associated with a higher risk of osteopenia and osteoporosis at a young age [70]. There is a complex interplay between insulin signaling, osteoblasts, and osteocalcin in glucose homeostasis. The activation of insulin receptors in osteoblasts modulates the synthesis of collagen [71]. Mice lacking insulin receptors in osteoblasts presented low levels of osteocalcin and reduced bone mineralization due to decreased bone formation and osteoblast development [72]. Insulin signaling stimulates osteoclast activity and therefore induces osteocalcin activation in osteoblasts [73]. Insulin also affects osteogenesis through an indirect mechanism by synergistic effects with other anabolic agents in bone, i.e., IGF-1 (Table 1, Figure 8) [74]. Higher levels of insulin growth factor-1 were associated with greater BMD and a decreased risk of fracture [75].

Serum hyperinsulinemia was associated with increased bone density [76], while the occurrence of insulin resistance in postmenopausal women without diabetes was associated with smaller bone size and greater volumetric BMD of the radius and tibia [77], as well as lower cortical bone volume and bone strength in the femoral neck [78].

The results of studies regarding the effect of insulin treatment on BMD are inconsistent. Dutta et al. presented that 1 year of insulin therapy was associated with a mild decrease in BMD at the hip [79]. The initiation of insulin in women with T2DM was related to a greater decline in BMD at the femoral neck [80]. Additionally, some studies show that patients with diabetes treated with insulin have an increased risk of fracture [81,82]. The increasing risk of nonvertebral fracture has even been found to be greater in men with T2DM who use insulin [83]. On the other hand, a case-control study of over 124 thousand patients with fractures shows a non-significant trend towards a reduced risk of fractures in insulin-treated T2DM patients [18]. Another study shows that the risk was lower in patients using long-acting insulins, which may be associated with the probability of insulin-induced hypoglycemia [84]. The long-acting insulin glargine has been reported to be associated with a reduced risk of fracture compared with an intermediate-acting insulin, which is usually the first insulin introduced to achieve adequate glycemic control. However, it is unclear whether this finding can be attributed to a reduced risk of hypoglycemia-related falls. To minimize the risk of fall-induced fracture, long-acting insulin might be the preferred treatment option for individuals at increased risk of hypoglycemia [85].

However, insulin treatment is often introduced in advanced stages of T2DM with an increase in the incidence of both microvascular and macrovascular complications, i.e., visual impairment or neuropathy, which may also contribute to falls and increase the risk of fractures [86], suggesting a broader combined effect of insulin treatment on bone metabolism. The incidence of hypoglycemia should be reduced by regular monitoring of blood glucose levels and appropriate education in diabetes self-management. By carefully assessing the insulin dose and thoroughly assessing potential causes of hypoglycemia, such as suboptimal timing or the site of insulin administration, renal and hepatic dysfunction, hypothyroidism, weight loss, and nutrition status, optimal glycemic control can be achieved. 

## 4. Conclusions

The relationship between BMD and T2DM is complex and involves a complex interaction of various factors. In T2DM, the risk of fractures can be higher even with normal or increased BMD due to reduced bone turnover, changes in bone quality, and impaired structure that alters biomechanical properties and therefore leads to bone fragility, often called the “diabetic bone paradox” [87]. Considering the pathophysiological mechanism, it is difficult to identify patients at-risk before the pathological fracture occurs because BMD measurements using traditional DXA and FRAX may often underestimate the fracture risk in diabetic individuals [88,89]. It may be worth using other available methods, such as trabecular bone score (TBS), quantitative computed tomography (QCT), volumetric BMD (vBMD), bone turnover biomarkers concentrations, or a combination of these. Future research is needed to address this clinical issue. 

This paper discusses the effects of various drugs used in the treatment of T2DM and on bone metabolism. Studies have tended to provide conflicting findings; however, TZDs, as the only category of drugs used to treat T2DM, appear to be associated with an increased risk of fractures and decreased bone mineral density, and should therefore be avoided in patients at risk of osteoporosis. However, many factors determine the final outcome, including the severity and duration of the T2DM, treatment, comorbidities, or glycemic control. In addition, fracture risk may also depend on hypoglycemia-induced falls, especially those related to insulin or sulfonylurea, as well as complications of T2DM. This hypothesis was confirmed in the most recent meta-analysis [90].

To minimize fall risk, it is necessary to implement regular strength and balance training programs, minimize environmental hazards, address individual medical causes and sensory impairments, and use assistive devices as needed [91]. Due to difficulties in differentiating the independent factors, we believe that pharmacotherapy for T2DM in patients should be assessed individually in terms of bone condition and glycemic control. Assessment of advanced glycation end products (AGEs), increased levels of reactive oxygen species (ROS), or cortisol release may increase the potential for future risk assessment or open new treatment pathways for patients with T2DM [6,92]. There are no specific recommendations for the treatment or diagnostic measures of osteoporosis in patients with diabetes, and current data on the impact of diabetes on bone metabolism are lacking and often controversial, which highlights the need for further investigation regarding the underlying mechanisms. Future research should investigate whether osteoporosis should be viewed as one of the natural complications in diabetic patients, alongside microvascular dysfunction, and whether current microvascular complications indicate patients at the highest risk of fracture. Assessment of low-energy fracture risk and screening osteoporosis should become a standard part of primary care for diabetes to prevent future skeletal complications. Moreover, the safety and effectiveness of antidiabetic drugs in patients with diabetes are also of particular interest for future research. 

## Figures and Tables

**Table 1:** Metabolic effects of antidiabetic drugs; symbols: ⬆—increase, ⬇—decrease.

Metformin	Sulphonylureas	SGLT-2 Inhibitors	DPP-4 Inhibitors	GLP-1 Agonists	Thiazolidinediones	Insulin
↓ intestinal glucose absorption	↑ insulin release by pancreatic B-cells	↑ urinary glucose excretion	↑ endogenous incretin concentration of GLP-1 and GIP	↑ GLP-1 receptor activation	↑ uptake of free fatty acids by adipocytes	↑ glucose utilization and storage by increasing glucose transport and net glycogen synthesis
↑ glucose utilization by intestinal cells	↑ tissue sensitivity to insulin	↑ glucose utilization	↑ sensitivity of pancreatic B-cells to glucose and ↑ glucose dependent insulin secretion	↓ glucagon secretion in a-cells	↑ secretion of adiponectin	↑ glucose transport into cells and net glycogen synthesis
↓ hepatic gluconeogenesis and glycogenolysis	↑ glucose transport into adipose tissue and muscles	↓ insulin resistance	↑ sensitivity of a-cells to glucose, glucagon secretion	↑ glucose-dependent insulin secretion	↓ production of TNF-a	white adipose tissue (WAT): ↓ lipolysis, ↑ glucose transport, ↑ lipogenesis
↑ glucose uptake and utilization by peripheral tissues	↑ glycogenesis in liver and muscles	↓ glucotoxicity	↓ hepatic glucose secretion both in fasting and postprandial states	↓ B-cell death, ↑B-cell proliferation, ↑ expansion of B-cell mass	↓ production of resistin	Liver: ↑ activation of glycogen synthesis, ↑ lipogenic gene expression, ↓ gluconeogenic gene expression
↑ peripheral insulin sensitivity	↓ synthesis of glucose and oxidation of fatty acids in the liver	adipose tissue: ↑ lipolysis, fatty acid oxidation and ketone body formation, ↓ visceral and epicardial fat mass	delay in gastric emptying, ↓ caloric intake and weight loss	↓ islet inflammation	↑ HDL-cholesterol concentration	Muscle cells: ↑ glycogenesis and ↓ protein synthesis, protein catabolism
↑ fatty acid oxidation in adipose tissue and skeletal muscles		Hepatic: ↑ gluconeogenesis, ↑ ketogenesis, ↑ hepatic glucose output, ↓ hepatic steatosis		↑ delayed gastric emptying, ↓ food intake, ↑ weight loss	↑ LDL-cholesterol concentration and particle size	Pancreatic beta cells: ↓ glucagon release
↑ lipolysis and inhibits lipogenesis		Cardiovascular: ↓ intravascular volume, ↓ blood pressure, ↓ cardiac preload and afterload, improves endothelial and ↓ vascular stiffness			↓ triglyceride concentration	
↓ the nuclear factor KB pathway in immune cells					↓ plasminogen activator inhibitor-1 and fibrinogen	
↓ the differentiation of monocytes to macrophages					anti-inflammatory effects	
↓ inflammation						

**Table 2:** Diabetes pharmacotherapy; summary of skeletal effects; symbols: ↑—increased; ↓—decreased; ↔—no change. Abbreviations: BMD = bone mineral density, T2D = type 2 diabetes.

Antidiabetic Medication	BMD	Fracture Risk	Overall Impact
Metformin	↑/↔	↓/↔	Most studies have shown beneficial effects on bone metabolism. Clinical data indicate neutral or even positive effects on bone and fracture risk, although metformin is usually used in individuals with a shorter history of diabetes with fewer complications.
Sulphonyloureas	limited data	↑ in at-risk individuals (elderly, frail, and post-menopausal women); results might be confounded by an increased risk of hypoglycemia-induced falls	Data on bone metabolism are very limited. Attention must be paid to the higher risk of hypoglycemia-induced falls.
SGLT-2 inhibitors	↔	↔, ↑ with canaglifozin	SGLT2 inhibitors are not significantly linked to an elevated risk of fractures; caution is advised with canagliflozin, which has raised concerns regarding potential detrimental effects on bone health.
DPP-4 inhibitors	↑, ↔	↔, ↓	DPP-4 inhibitors have been reported to have neutral or beneficial effects on bone by the majority of studies and have been associated with a lower incidence of fractures.
GLP-1 receptor agonists	↔	↔	Preclinical models show a beneficial effect on bone. Clinical data show mostly neutral effects, although a few studies have shown harmful or beneficial effects on risk for fracture.
Thiazolidinediones	↓	↑	There are negative effects on bone metabolism and an increase in fracture risk.
Insulin	↑	↑ in T2D	Insulin use in T2D is associated with ↑ fracture risk. Maintenance of tight glycemic control should be avoided due to increased episodes of hypoglycemia, falls, and fractures in at-risk populations.

## Data Availability

Not applicable.
